# Droplet digital PCR application for the detection of SARS-CoV-2 in air sample

**DOI:** 10.3389/fpubh.2023.1208348

**Published:** 2023-10-27

**Authors:** Siti Aishah Rashid, Raheel Nazakat, Rosnawati Muhamad Robat, Rohaida Ismail, Jeyanthi Suppiah, Kamesh Rajendran, A. S. Santhana Raj Louis Masalamany, Nur Afrina Muhamad Hendri, Nadia Mohamad, Nurul Amalina Khairul Hasni, Fatin Amirah Suib, Nik Muhamad Nizam Nik Hassan, Muhammad Alfatih Pahrol, Rafiza Shaharudin

**Affiliations:** ^1^Environmental Health Research Centre, Institute for Medical Research, National Institutes of Health, Ministry of Health Malaysia, Shah Alam, Selangor, Malaysia; ^2^Infectious Disease Research Centre, Institute for Medical Research, National Institutes of Health, Ministry of Health Malaysia, Shah Alam, Selangor, Malaysia; ^3^Special Resource Centre, Institute for Medical Research, National Institutes of Health, Ministry of Health Malaysia, Shah Alam, Selangor, Malaysia

**Keywords:** SARS-CoV-2 RNA, air sample, droplet digital PCR, specificity, sensitivity

## Abstract

Severe Acute Respiratory Syndrome Coronavirus 2 (SARS-CoV-2) may transmit through airborne route particularly when the aerosol particles remain in enclosed spaces with inadequate ventilation. There has been no standard recommended method of determining the virus in air due to limitations in pre-analytical and technical aspects. Furthermore, the presence of low virus loads in air samples could result in false negatives. Our study aims to explore the feasibility of detecting SARS-CoV-2 ribonucleic acid (RNA) in air samples using droplet digital polymerase chain reaction (ddPCR). Active and passive air sampling was conducted between December 2021 and February 2022 with the presence of COVID-19 confirmed cases in two hospitals and a quarantine center in Klang Valley, Malaysia. SARS-CoV-2 RNA in air was detected and quantified using ddPCR and real-time reverse transcriptase-polymerase chain reaction (RT-PCR). The comparability of two different digital PCR platforms (QX200 and QIAcuity) to RT-PCR were also investigated. Additionally negative staining transmission electron microscopy was performed to visualize virus ultrastructure. Detection rates of SARS-CoV-2 in air samples using ddPCR were higher compared to RT-PCR, which were 15.2% (22/145) and 3.4% (5/145), respectively. The sensitivity and specificity of ddPCR was 100 and 87%, respectively. After excluding 17 negative samples (50%) by both QX200 and QIAcuity, 15% samples (5/34) were found to be positive both ddPCR and dPCR. There were 23.5% (8/34) samples that were detected positive by ddPCR but negative by dPCR. In contrast, there were 11.7% (4/34) samples that were detected positive by dPCR but negative by ddPCR. The SARS-CoV-2 detection method by ddPCR is precise and has a high sensitivity for viral RNA detection. It could provide advances in determining low viral titter in air samples to reduce false negative reports, which could complement detection by RT-PCR.

## 1. Introduction

COVID-19, a potentially deadly disease caused by the Severe Acute Respiratory Syndrome Coronavirus-2 (SARS-CoV-2), quickly became a global public health crisis due to its emergence and rapid spread (1). As of September 2022, there were 612 million confirmed cases reported globally with 6.5 million deaths (2). It is notable that SARS-CoV-2 has been primarily transmitted via respiratory droplets of various sizes (3–5). Additionally, it has also been declared as an airborne disease (6, 7).

If a person nearby is displaying respiratory symptoms like coughing or sneezing, it is possible for the virus to spread through larger respiratory droplets (>5 μm) (8, 9). In contrast, smaller respiratory droplets (≤ 5 μm) containing the virus can remain in the air for extended periods and travel a greater distance of more than 6 meters, as seen in the cases of tuberculosis, measles and chickenpox (10, 11).

Further understanding on other mechanism of SARS-CoV-2 transmission, especially airborne route, poses critical roles of healthcare response that will help in putting appropriate safeguards to protect public's health (12–14). At the beginning of the COVID-19 outbreak, there was a discussion regarding the potential airborne transmission of SARS-CoV-2. Nevertheless, recent research on aerosols have demonstrated the detection of SARS-CoV-2 ribonucleic acid (RNA) in hospital rooms and isolation wards where COVID-19 patients are being treated. The virus is detectable in air samples and the detection is achieved by synthesizing complementary deoxyribonucleic acid (cDNA) and subsequent polymerase chain reaction (PCR) to amplify specific sequences of the SARS-CoV-2 genome.

The gold standard for detection of the virus in clinical samples is RNA amplification techniques, as recommended by the World Health Organization (WHO) and the Center for Disease Control and Prevention (CDC) (15, 16). Nevertheless, the reverse transcriptase Polymerase Chain Reaction (RT-PCR) has technical pitfalls which includes susceptibility to inhibitors, low amplification efficiency, showing poor detection in samples with low concentration, relying on subjective cut-off values, and requiring standard curve-based quantification (17, 18). Digital droplet PCR (ddPCR) has been utilized to detect low concentrations sample, as it enables absolute quantification and serve as a precise alternative to conventional PCR methods (19). As such, ddPCR has been incorporated into clinical studies of SARS-CoV-2 to identify false-negative biological samples that were obtained through conventional RT-PCR (20). Given that the concentration of the virus in air samples is anticipated to be considerably lower than in biological samples, utilizing ddPCR for the identification of SARS-CoV-2 in bioaerosols may serve as a preferable alternative.

In this regard, our study aimed to establish ddPCR as a better method for the detection of SARS-CoV-2 in air samples and to test the sensitivity and specificity of ddPCR compared with RT-PCR. We also proposed optimized parameters for better sampling and detection of SARS-CoV-2 viruses in air samples.

## 2. Materials and methods

Air samples were collected between December 2021, and February 2022 in two hospitals and a quarantine center in Klang Valley, Malaysia with the presence of COVID-19 confirmed cases. To minimize the collection of negative samples we only select patients with confirmed PCR test within 5 days and RT-PCR equal or <38 (Ct ≤ 38) were included in this study.

### 2.1. Air sampling

**Figure 1:**
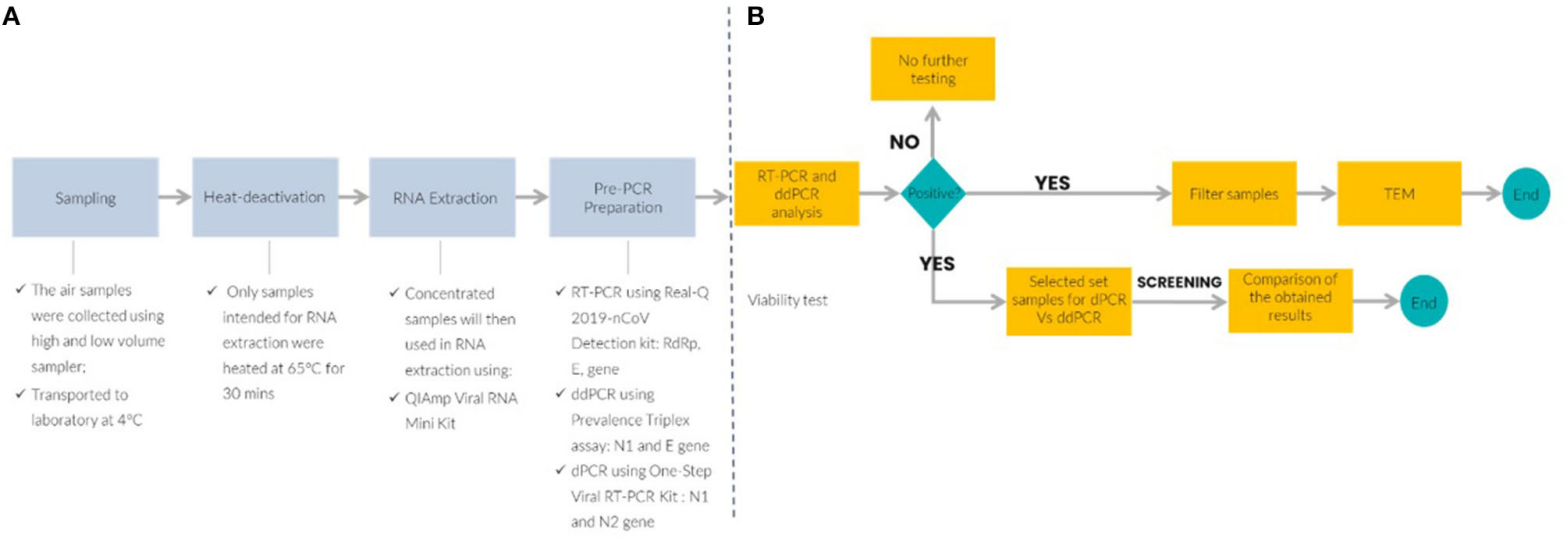
Schematic workflow of the experimental air sampling set-up and molecular detection platform. **(A)** Upstream process to detect SARS-CoV-2 in air samples and **(B)** downstream analysis for SARS-CoV-2 detection by RT-PCR, reverse transcription quantitative polymerase chain reaction; ddPCR, droplet digital PCR; TEM, transmission electron microscope; dPCR, digital PCR.

Sample collection was performed in two designated COVID-19 hospitals and a quarantine center. These locations include medical wards, ICU, three quarantine center halls, and emergency department. The locations were ventilated with either air handling unit (AHU) or frequency conversion technology (FCT). This study utilized both active and passive air sampling methods. The air sampling approaches were as follows: (i) passive sampling using a settle plate (5 mL, SKC Inc., Eighty-Four, PA, USA), and (ii) active sampling using NIOSH BC-251 bio-sampler (Morgantown, WV, USA), and Coriolis (cyclone) (Grade EPM 2000, 47 mm, Whatman^®^, USA). Passive sampling involves the placement of culture plates, containing viral transport media (VTM), with opened lids for 4 h (21) at the height of one-meter above the ground level while maintaining at least a one-meter distance from any physical barriers such as walls, windows, entrance doors or any surrounding obstacles. NIOSH BC-251 bio-samplers were used to collect aerosols at a flow rate of 3.5 L/min for 4 h (22–24). Coriolis^®^ collected consecutive air samples for 10 min each with an airflow rate of 100 L/min (total of 1 m^3^). Besides collecting air samples, a set of control samples were collected on each sampling day by placing the sample tube or filter paper into the machine without collecting the air, prior to the air sampling. Control samples were treated the same as air samples from the patient care areas. The overall workflow of the experimental set-up is shown in Figure 1.

### 2.2. Sample preparation

Membrane filter from the NIOSH BC-251 bio-samplers was processed according to Thedell et al. (25). The filter was extracted from the cassette using sterile forceps and then divided into four parts. Each part was immersed in a separate tube containing 5 mL of sterile RNase-free water. To remove viral particles that may be attached to the membrane, the tubes were shaken at 250 revolutions per minute (RPM) for an hour. It was then centrifuged at 21,913 RCF for 5 minutes (min) to eliminate any debris. The supernatants were transferred to new microcentrifuge tubes for viability testing, viral nucleic acid extraction, and electron microscopy. After a 4-h sampling period, samples collected from NIOSH BC-251 bio-samplers were processed as follows: 1 mL Viral Transport Medium (VTM) was added to the 1.5 mL tube, inverted, and vortexed several times before transferring the 1 mL VTM to the 15 mL tube, again the tube was inverted, vortexed and finally proceed for downstream analysis. On the other hand, air samples from the Coriolis^®^ were collected on a wet medium, with 5 mL of sterile VTM added to the collection cones prior to sampling.

### 2.3. RNA extraction and concentration

Before proceeding with RNA extraction, the samples were subjected to a heat treatment at 65°C for 30 min. The QIAamp^®^ Viral RNA Mini Kit (Qiagen, Hilden, Germany) was used with some modifications to the manufacturer's instructions. A larger input samples volume of 420 μl was used, and the amounts of lysis buffer and other reagents added were increased proportionally. However, the volumes of Buffers AW1 and AW2 used for the washing steps remained unchanged. The extracted RNA was eluted (80 μl) using a 2 X 40 μl Buffer AVE and the resulting RNA was stored in duplicate at −80°C until further amplification.

### 2.4. Molecular viral load quantification

#### 2.4.1. Real-time RT-PCR

The Real-Q 2019-nCoV Detection Kit (BioSewoom, Seoul, Republic of Korea) was used to perform real-time RT-PCR amplification of SARS-CoV-2. A 25 μl reaction mix was prepared, consisting of 8.5 μl of extracted RNA, 1.0 μl of RT-PCR enzyme, 12.5 μl of 2X PCR mixture, and 3.0 μl of nCoV probe and primer mixture. The probe detection modes were set as SARS-CoV-2 RNA-dependent RNA polymerase (RdRp) gene using 6-carboxyfluorescein (FAM), Beta nCoV E gene using Hexachloro-fluorescein (HEX) and Internal Control using Cyanine-5 (Cy5). Positive and negative controls were included each run with samples. Positive control (PC) for real-time RT-PCR is RdRp, E and HRP gene at a concentration of 160x LOD according to the manufacturer product insert and negative control (NTC) is nuclease free water. The PCR amplification was carried out on a CFX Opus 96 thermal cycler (BioRad, Munich, Germany) under the following conditions: (i) denaturation for 30 min at 50°C, (ii) annealing for 15 min at 95°C, (iii) 40 cycles of 95°C for 15 seconds (s) and extension at 62°C for 45 s. To be valid all positive samples should exhibit fluorescence amplification curves for RdRp gene (FAM channel) and E gene (HEX channel) with cycle threshold (Ct) lower or equal to 38 cycles (≤ 38). The result with Ct lower or equal to 38 cycles (≤ 38) for only E gene is defined as presumptive positive. Ct values beyond 38 cycles (>38) were considered negative. An internal control detection was included in the Cy5 channel.

#### 2.4.2. RT-ddPCR

Absolute quantification of SARS-CoV-2 RNA in aerosol samples was performed using the 2019-nCoV CDC ddPCR Triplex Probes and the One-Step RT-ddPCR Advanced Kit for Probes (Bio-Rad, USA). The CDC N2 and E primer-probes sets were utilized for this purpose. The PCR mixture was standardized with 20% overage per sample based on the manufacturer's recommendation. The reaction mixture (24 μl) consisted of RNA template (10.8 μl), one-step supermix (6.0 μl), one-step reverse transcriptase (2.4 μl), 300 mM DTT (1.2 μl), ddPCR triplex assay (1.2 μl), and nuclease-free water (2.4 μl). A pre-PCR room was used for preparing the reaction mixtures to minimize reagent contamination. As per the manufacturer's guidelines, a total volume of 20 μl of reaction mixture was utilized to produce droplets using the Bio-Rad Droplet Generator. Positive and negative controls were included each run with samples. Positive control (PC) for ddPCR is N2, and E gene and negative control (NTC) is nuclease free water. Prior to amplification, the emulsion resulting from this process was transferred to a new 96-well plate (Cat No. 951020389, Eppendorf, Enfield, CT). Subsequently, PCR amplification was carried out in a C1000 (Bio-Rad, USA) thermocycler. The amplification process comprised of the following conditions: (i) reverse transcription (RT) step at 50°C for 60 min, (ii) polymerase activation at 95°C for 10 min, and (iii) 40 cycles of denaturation at 94°C for 30 s, followed by annealing/extension at 55°C for 60 s. Subsequently, polymerase deactivation was performed at 98°C for 10 min and the droplets were stabilized at 4°C for 30 min before being read using a QX200 droplet reader (Bio-Rad, USA). The absolute quantification was performed using QX Manager Software Standard Edition, v 1.2. Samples with an average number of accepted droplets <10,000 were considered non-quantifiable for analysis. For N2 and E primers/probe sets, the positive threshold for detecting SARS-CoV-2 RNA through ddPCR was determined to be 0.07 copies/μl or higher. An outcome of below 0.07 copies/μl for both N2 and E primers/probe sets was interpreted to be negative. This value is determined through the analysis of multiple negative controls and the evaluation of their fluorescence amplitude levels. Threshold 0.07 copies/μl that can effectively distinguish true positive signals from background noise were selected.

#### 2.4.3. dPCR

dPCR was carried out on selected sub-samples using optimized CDC N1 and N2 assays. QIAcuity One-Step Viral RT-PCR Kit (Catalog No. 1123145, Qiagen) and 26000 24-well Nanoplates (Catalog No. 250001, Qiagen) were used to perform the experiment. An amount of 40 μl of reaction mixtures were prepared in a 96-well preplate and consists of QIAGEN 4X One-Step Viral RT-PCR Master Mix (10 μl), QIAGEN 100X Multiplex Reverse Transcription Mix (0.4 μl), GT-Molecular 20X assay solution (2 μl), DNase and RNase free water (7.6 μl), and template RNA (20 μl). Positive and negative controls were included each run with samples. Positive control (PC) for ddPCR is N1, and N2 gene and negative control (NTC) is nuclease free water. Upon completing the transfer of the reaction mixture into the 26000 24-well Nanoplates, it was loaded onto the QIAcuity dPCR 5-plex platform (Qiagen). An automated workflow occurs in the instrument in which each sample was partitioned into 26000 partitions, followed by PCR reactions on each of the partition. The following protocols were carried out for PCR amplification: (i) 50°C for 30 min for reverse transcription, (ii) 95°C for 2 min for enzyme activation, (iii) 95°C for 10 s for denaturation, and (iv) 45 cycles of annealing/extension at 55°C for 30 s, and a final imaging in the FAM channel. RT-dPCR controls were included in the experiments, consisting of negative and positive (γ-irradiated SARS-CoV-2 RNA) controls. The QIAcuity Suite Software version 2.1.7.182 (Qiagen) was used for data analysis, and the quantities were reported as GC per microliter of reaction mixture. To minimize errors, automatic settings were used for the threshold and baseline during the RT-dPCR assays (26). Any values above 0 copies/μl were considered as positive.

### 2.5. Transmission electron microscope

Immediately after stripping process from the membrane filter as mentioned previously, samples collected in nuclease free water (NFW) were fixed with 2.5% glutaraldehyde in 1.0N phosphate buffer solution (PBS) at room temperature for at least 1 h. After fixation, viral particles were concentrated using Airfuge (Beckman, USA) at 90 000 RPM for 15 min and stained with 1% ammonium molybdate for 1 min. In-house SARS-CoV-2 culture processed with negative staining TEM is regarded as positive control. For negative control, a set of control samples were collected on each sampling day by placing the filter paper into the machine without collecting the air, prior to the air sampling. Control samples were treated the same as air samples from the patient care areas. Negative controls were determined by screening through negative staining TEM, viral undetected. The samples were screened for viral analysis under TEM Tecnai G2 Spirit Twin (Thermofisher Scientific, Waltham, MA, USA) with 120 kilovolts (kV). Images were digitally recorded and analyzed using an embedded control CCD camera (Gatan, California, USA).

### 2.6. Sensitivity and specificity analysis

The analytical sensitivity and specificity were calculated based on the detection of SARS-CoV-2 RNA, in which to assess the reliability of both dPCR platforms. The ability of the index test to correctly identify positive results is referred to as sensitivity and is calculated using [Eq. (1)]. Specificity, on the other hand, is used to eliminate false negative results and is calculated using [Eq. (2)].


(1) $$\begin{array}{l} Sensitivity=\;\frac{True\;Positives\;\left(A\right)}{True\;Positives\;\left(A\right)+False\;Negatives\;\left(C\right)} \end{array}$$

(2) $$\begin{array}{l} Specificity=\;\frac{True\;Negatives\;\left(D\right)}{True\;Negatives\;\left(D\right)+False\;Positives\;\left(B\right)} \end{array}$$

### 2.7. Ethics statement

The study protocol was evaluated by the Medical Research and Ethics Committee (MREC), Ministry of Health Malaysia (MOH). As the study did not fall within the scope of the Malaysian Act on Medical Research Involving Human Subjects no further medical ethical approval was required. Ethical approval of this study was received from the Medical Research and Ethics Committee [KKM/NIH/P21-1159 (4)]. Throughout the research, all methods were carried out with the highest ethical considerations and in accordance with relevant guidelines and regulations—Guidelines for Conducting Research in Ministry of Health Institutions and Facilities.

## 3. Results

### 3.1. SARS-CoV-2 nucleic acid detection by ddPCR, RT-PCR, and TEM in air samples

**Figure 2:**
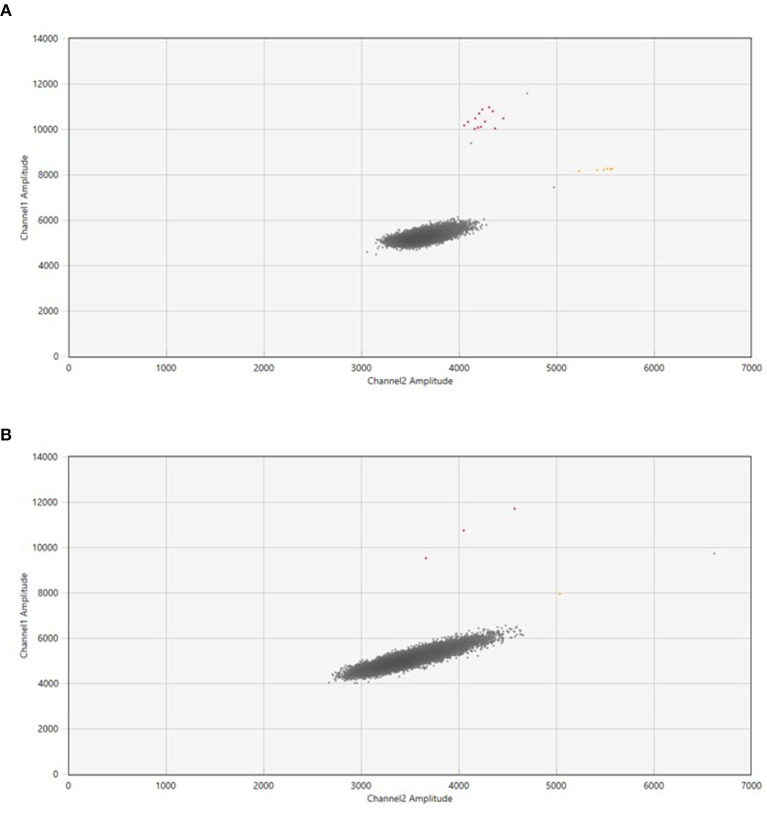
The QuantasoftTM Software (Bio-Rad Laboratories) generated ddPCR two-dimensional scatterplots for the SARS-CoV-2 RNA in air samples. **(A)** Showed samples with a high number of droplets in the FAM and FAM+HEX-channel, while **(B)** displayed fewer droplets in both channels in contrast to the number of droplets in **(A)**. The X-axis represented the fluorescence intensity in the HEX-channel (channel 2), while the Y-axis represented the fluorescence intensity in the FAM-channel (channel 1). The background fluorescence is represented by the gray dots, the fluorescence detected in the FAM+HEX-channel (E gene) is represented by the yellow dots, and the fluorescence detected in the FAM-channel (N2 gene) is represented by the red dots.

The number of samples collected were 167, consisting of 145 samples collected through active sampling (LVS-NIOSH: 101 and HVS-Coriolis: 44) and 22 samples collected through passive sampling (settle plates). Out of 101 samples collected using high volume sampler, 55 samples were collected in tubes while 46 samples were trapped in the filters. The collected air samples from hospitals and quarantine center were tested using 2 platforms, ddPCR and RT-PCR. To avoid false positives due to contamination, the experiments were performed inside a biosafety cabinet. Detection rate by ddPCR on air samples collected through active sampling approach was 15.2% (22/145) and only 3.4% (5/145) by RT-PCR. The ddPCR negative values for N2 and E genes ranged from ND – 0.069 copies/μl primers/probe sets respectively (Figure 2). RT-PCR Ct values in negative samples for RdRp and E gene range from 39 to 39.82 and to 39.40 respectively (Table 1).

**Table 1:** Specificity and sensitivity evaluation for SARS-CoV-2 detection using two different digital PCR system with reference to RT-PCR as the current gold standard method.

**Platform**	**Positive detection rate (%)**	**Sensitivity (%)**	**Specificity (%)**
dPCR (QIAcuity)	26.47 (9/34)	50.00	76.67
ddPCR (QX200)	38.23 (13/34)	75.00	66.67

ddPCR analysis were done immediately after RNA extraction while dPCR analysis were done 2 months after RNA extraction.

**Figure 3:**
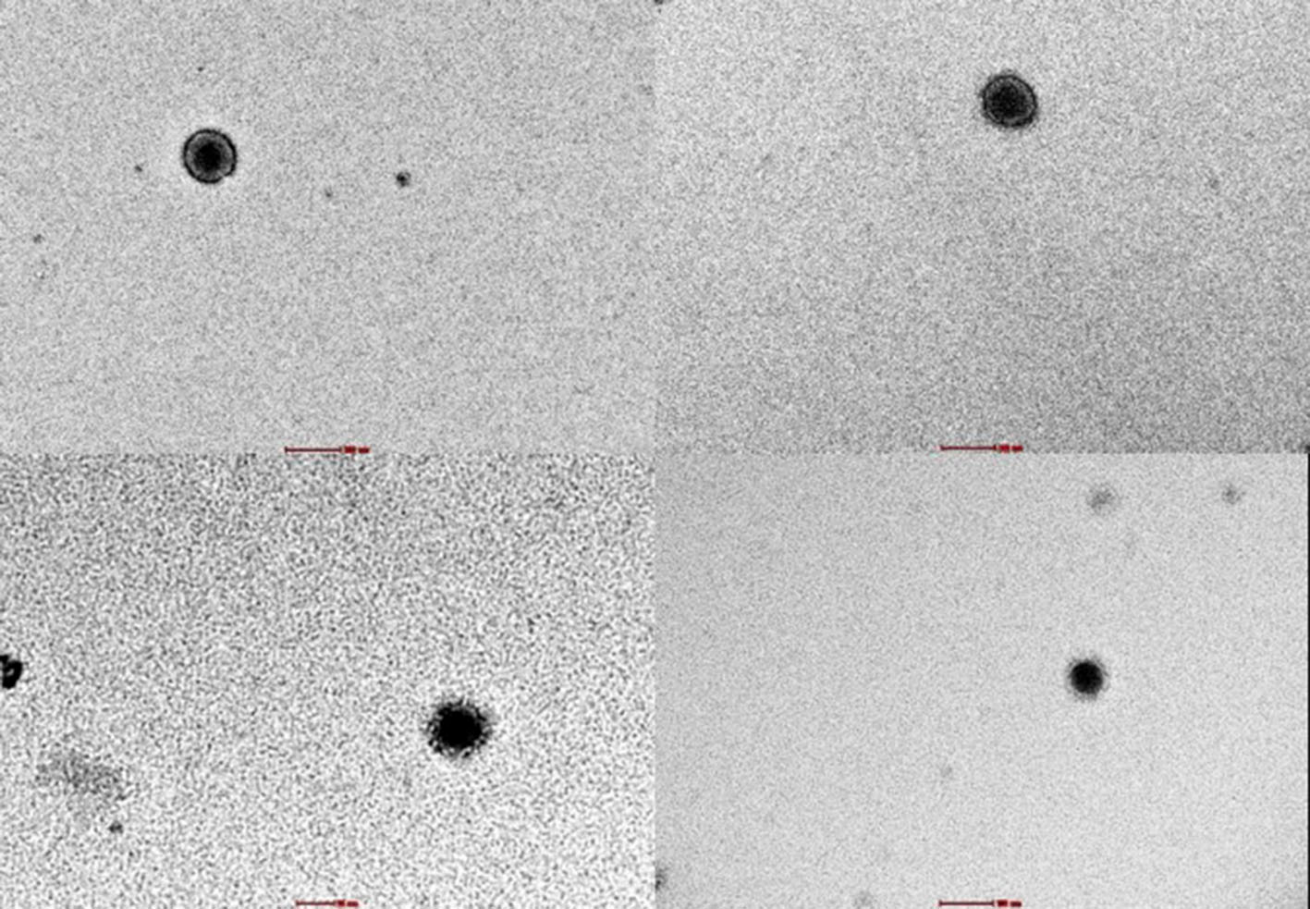
Viral with capsid were seen under negative staining through TEM in 18,000x magnification. These identifications were confirmed with ddPCR.

**Figure 4:**
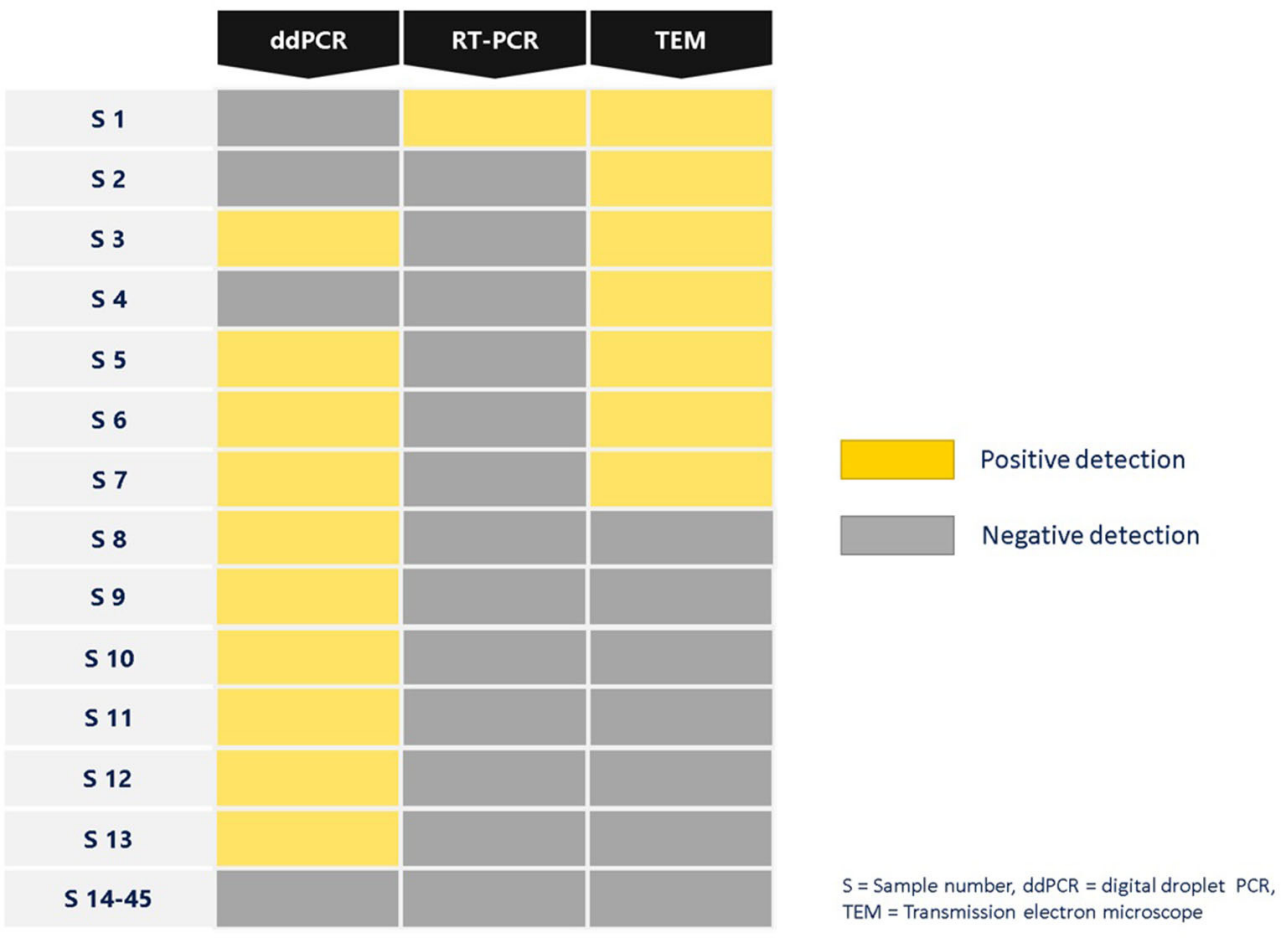
Summary of SARS-CoV-2 detection in air samples isolated from air sampler membrane filter using different platforms ddPCR, RT-PCR and TEM.

The examination of SARS-CoV-2 ultrastructure was carried out using negative staining TEM after exposing the sample to a solution phase, following the method outlined by Prasad et al. (27). The aim was to detect the presence and characterize the nature of morphology seen in the virus particle from air samples retrieved from membrane filters (Figure 3). Through TEM, 15.55% (7/45) tested positive and out of these, 8.88% (4/45) were detected by both TEM and ddPCR (Figure 4). The morphodiagnostic characteristics of seven negative-stained coronavirus-like in the scanned fields has a size with range 70 nm-−90nm and spherical form. The virus is depicted as being round, with an envelope, capsid, and spikes that resemble crowns. One specific viral particle was exceptionally well-preserved and had coronaviruses' typical morphodiagnostic characteristics. This 75 nm-sized particle had irregular stain pooling on its surface and a characteristic envelope projection that terminated in circular “peplomeric” structures (Figure 4).

### 3.2. Comparison of SARS-CoV-2 specificity and sensitivity by RT-PCR and ddPCR

Considering the results of SARS-CoV-2 detection from both RT-PCR and ddPCR, it was noted that ddPCR displayed higher accuracy and robustness in comparison to RT-qPCR. The results further confirmed the high sensitivity (100%) of ddPCR in detecting target gene in air samples. In contrast the specificity for ddPCR was reported to be slightly lower at 87.14%. Partitioning through digitization, in particular, appears to decrease the susceptibility to traditional PCR inhibitors. Research by Dingle et al. (28) support this phenomenon and its underlying mechanism.

### 3.3. Assessment of analytical sensitivity and specificity for SARS-CoV-2 detection using two different digital PCR platforms

**Figure 5:**
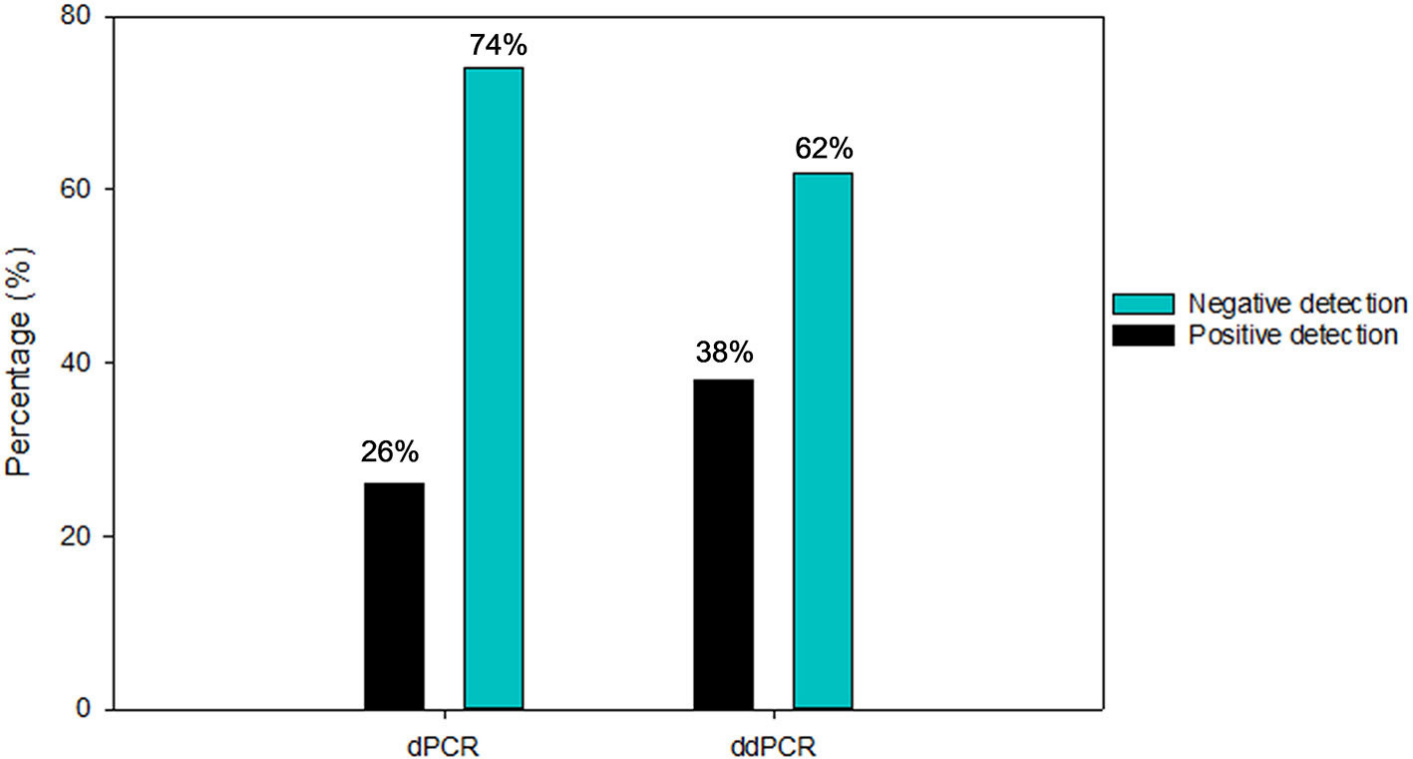
Number of positive and negative air samples detection from diagnostic performance for SARS-CoV-2 detection using ddPCR and dPCR platforms.

Out of 145 air samples collected, thirty-four air samples were used for the evaluation sample set between the two digital PCR platforms. These samples were previously tested positive (*n* = 4) and negative (*n* = 30) by RT-PCR. The first system by ddPCR (BioRad QX200), 62% (21/34) of the samples were reported to be negative, while 38% (13/34) positive reported as positive. The follow up analysis by dPCR (QIAcuity) revealed that 74% (25/34) were reported as negative, and 26% (9/34) with positive detection (Figure 5). dPCR detection for N1 gene range from ND-0.57 copies/μl and N2 gene range from ND −0.39 copies/μl primers/probe sets respectively, while detection for N2 gene range from ND-0.31 copies/μl and E gene range from ND −1.30 copies/μl primers/probe sets, respectively, for ddPCR.

The results were compared to RT-PCR and confirmed the higher specificity 76.67% of dPCR but a slightly lower sensitivity (50%) in detecting SARS-CoV-2 viral RNA in air samples. In contrast, the sensitivity and specificity for ddPCR for the same set of air samples specimens was reported to be 75.00 and 66.67%, respectively (Table 1). Excluding the 17 samples (50%) that were negative by both ddPCR and dPCR, 5 samples (15%) were both ddPCR and dPCR positive. There were 23.5% (8/34) samples that were detected positive by ddPCR but negative by dPCR. In contrast, there were 11.7% (4/34) samples that were detected positive by dPCR but negative by ddPCR.

### 3.4. Parameters optimization for SARS-CoV-2 detection in air sample

Overall, a few other parameters have been optimized in order to monitor the presence of SARS-CoV-2 particles in air samples. Among the parameters included air sampling approach, samples concentration, and sampling buffer for electron microscopy. The performance of passive and active air sampling approaches was also evaluated. This included using active air sampler (LVS and HVS), and passive settling plates to detect SARS-CoV-2 RNA virus and comparing detection efficacy among sampling methods. Out of the 211 samples collected, 44 were controls and all of them tested negative. The detection rate of SARS-CoV-2 in the collected samples was 15% (22/145) using active air samplers (LVS and HVS), compared to 9.1% (2/22) detection by passive air sampling. Six of the positive samples with the detection rate of 13.6% (6/44) were collected using HVS. Positive detection rate for LVS was 15.84% (16/101). A total of 4.9% (5/101) of positive detection rate samples were collected from the 1.5 mL and 15 mL tubes using LVS Bio-sampler with particles size range between 1 to more than 4 μm in diameter. The most positive samples collected were on filter, with a detection rate of 10.89% (11/101). The RNA extraction procedure was optimized to increase concentration of RNA virus recovery from air samples. Using 1X (140 μl) sample volume results in no positive detection by both RT-PCR and ddPCR. Increasing the volume of air samples to 2X (280 μl) increased the positive detection rate of ddPCR by 1.4% (2/145). The 3X sample volume (420 μl) increases the positivity rate by 11-fold (15/245).

## 4. Discussion

The study compared sensitivity and specificity of RT-PCR (Bio-Rad CFX96) and ddPCR (BioRad Qx200) techniques in detecting SARS-CoV-2 viral RNA in air samples at areas treating COVID-19 confirmed cases. The advantages and limitations of two methods for evaluating potentially critical false-negative samples were compared by analyzing air samples collected from hospitals and quarantine centers. By RT-PCR, detection rate results (3.4%) demonstrated that, it is insufficient to identify positive air samples with low viral loads. The probe showed that the assay chemistry allows the identification of the positive sample only at a very late Ct value (36.08–38.00). Samples that exhibit a Ct value of more than 38 are often deemed unsatisfactory due to their suboptimal amplification characteristics. In such cases, these samples need to be repeated to evaluate whether they are true positive or a false positive (29). False negative results from the real-time RT-PCR assay can occur as a result of inadequate viral traces in the samples, whereas weak positive results (i.e. high Ct values) may be challenging to decipher and distinguish from artifacts such as probe degradation or increase in fluorescence due to background noise and others (30).

To resolve this situation, we found ddPCR allowed to diagnose with certainty the tested sample as positive at very low RNA concentration. The detection limit for the N2 and E gene is lower than 0.1 cp/μl. According to Freire-Paspuel et al. (31), RT-PCR assay has a detection limit of 5–10 copies/μl. Due to its high sensitivity in detecting and quantifying target RNA (32, 33) and can deliver accurate results even at considerably low viral titer, ddPCR recently is regarded as a good substitute for RT-PCR assays (34). The ddPCR technology is frequently employed to detect circulating tumor DNA, but its application to infectious diseases, particularly viruses, has not received as much attention. Examples of viruses that have been detected by ddPCR include influenza virus, hepatitis B virus (HBV), human papillomavirus (HPV), human immunodeficiency virus (HIV), and cytomegalovirus (35–41). This technique has been used to measure the copy number of HBV DNA (35), HIV RNA for viral load (40) and it also shows high sensitivity in detecting influenza A virus, human papillomavirus DNA in cytology and formalin-fixed specimens (42). Several articles have indicated that ddPCR assays have enhanced sensitivity and specificity compared to RT -qPCR for the identification of SARS-CoV-2 infection, specifically in samples with lower viral loads (20, 43–45). Previous studies have shown greater sensitivity and specificity as compared to other methods, such as qPCR and serological tests (35). Our research confirmed the findings of earlier studies and showed that ddPCR had a higher diagnostic potential than RT-PCR.

When negative-stained TEM and the PCR tests were used in parallel, TEM results acted as a complementary method to molecular methods and explained some differences detected in PCR tests. Positive samples obtained by both ddPCR and TEM are more than positive samples obtained by both RT-PCR and TEM. In Figure 4, positive samples obtained by ddPCR are negative in the TEM assay can be due to the fact that the SARS-CoV-2 should be encapsidated and structurally intact in order to be able to be visualized using TEM. In addition TEM imaging was limited by particle load in the specimen which is the main issue with air samples. In the case of positive samples obtained by TEM resulted in negative by ddPCR, can be explained in terms of variations in sensitivity, sample preparation, genetic variation, inhibitors/contaminants, and experimental error could contribute to positive SARS-CoV-2 air samples appearing negative by ddPCR. Through EM, the air samples were shown to have the morphology of a typical coronavirus (SARS-CoV-2). It is an enveloped particle with a size variation of 75 to 200 nm. It has a distinct fringe of club-shaped peplomers, as seen from a TEM (46, 47). Comparison of results from RT-PCR and ddPCR shows that ddPCR will be an important tool for detecting SARS-CoV-2 in air samples.

The technique of digital PCR is exceptional due to its ability to quantify the precise copy number of a target DNA, without the need for external standards. Comparability across various dPCR platforms, however is scarce. In the study presented here, we compared dPCR (QIAcuity) and ddPCR (BioRad QX200) platforms for the sensitivity and specificity in detecting low SARS-CoV-2 RNA concentration in air samples with emphasis on the comparability between platforms. There were studies that previously evaluated the performance of various digital PCR platforms for accurate quantification of DNA copy number of a certified plasmid DNA reference material (48). The dPCR was the least sensitive of the two systems with a lower sensitivity (38%) than the ddPCR (56%). This basically explain that both platforms lose the ability to pick up the detection of SARS-CoV-2 about 60% by dPCR and 40% by ddPCR, respectively. These results provide an independent evaluation of methods for determining RNA copy number using different dPCR platforms and highlight important factors, such as dead volume, that should be considered when performing dPCR experiments. Dead volume refers to the portion of samples that are not compartmentalized or analyzed. Since all digital PCR techniques required compartmentalization (e.g.,: droplets, nanoplates, crystals etc) there could be some portion of samples that are lost along the process. The lost portion for QIAcuity 26K partitions was 46% and for BIORAD 25K droplet partitions it was ~43–48%. Thus, it is important to ensure the complete total volume of reaction in order to calculate the number of total valid partitions and total analyzable volume. QIAcuity 26K nanoplate reaction volume was 40 μl. It allows for the analysis of 21.6 μl which results in 18.4 μl (46%) of dead volume. On the other hand, the reaction volume for BIORAD ddPCR 25k droplets is 20 μl and the dead volume is between ~9.4–10.5 μl (~43–48%) (49). Considering 43% as the dead volume for BIORAD QX200, it fits with our finding that BIORAD system offers a better sensitivity. However, other limitation of this study was the timing of analysis. Samples tested by dPCR was conducted 2 months after RNA extraction compared to analysis by RT-PCR and ddPCR which were done immediately after RNA extraction process. The extracted RNA tested by dPCR might be susceptible to degradation during the time the analysis was conducted.

Other study also highlighted that SARS-CoV-2 detection in air is challenging, and differences in the air sampling method may affect sampling efficiency and virus detection (50). As such, several parameters were also optimized, including (i) sample volume for extraction, (ii) air sampling approach and (iii) sampling buffer for electron microscopy to improve the SARS-CoV-2 detection in air samples. In this study, we discovered that detection rate by active air samplers was 165% more than passive sampling. This suggests that active air sampler was even more robust for identification of SARS-CoV-2 in air compared to passive air sampling. Detection efficiency is reduced in passive sampling because it relies on the natural forces to deposit bioaerosol particles on a collection medium (51).

The benefits of employing larger sample volumes in the extractor to boost the sensitivity of RT-PCR-based amplification methods for the detection and quantification of viruses have been emphasized by a number of studies (52–54). Our study confirmed these advantages for the detection of SARS-CoV-2, as the sensitivity of the ddPCR was enhanced 11-folds by using 420 μl of sample in the extraction compared to smaller volume of 140 μl. When sterile NFW, instead of VTM, was used as a buffer for TEM analysis, it gives a better visualization of the SARS-CoV-2 structure/morphology. VTM contains Hanks Balanced Salt Solution (HBSS) with 1X calcium and magnesium ions (55). The calcium and magnesium ions are reducing agents that may affect the staining quality for electron microscopy visualization (56).

### 4.1. Strength and limitation

This study is limited by units in which the usage of both quantitative (copies/μl) and qualitative (Ct value) detection approach with different primer probes for both approaches were compared. In addition to that differences in other PCR components for instance, the use of different reverse transcriptases, polymerases and number of cycles can also affects the detection of airborne SARS-CoV-2 (57). Secondly, there is also no consistence guidance on the better usage of either LVS or HVS in sampling strategy due to types of air sampler used. Various papers presented inconsistent and did not allow any meta-analysis for better decision. This study however able to compare which air samplers give better results. Although statistically there is no significant different in detection through LVS and HVS, but we are able to report that detection rate was highest for LVS through membrane filter compared to other samplers. Third, despite the fact that ddPCR is more sensitive than RT-PCR at detecting SARS-CoV-2 in the air samples used in this work, there are still a number of drawbacks. In contrast to RT-PCR, the cost of the BIORAD Q200 ddPCR and QIAquity dPCR platforms and related consumables is higher. Finally, ddPCR experiments often take longer (about 4–5 h) than RT-PCR experiments (around 2 h). However, the limitation may be overcome by QIAquity platforms, that require almost similar time to RT-PCR which is about 2 h experiments. Notwithstanding these drawbacks, the study findings provided here indicate that ddPCR, as compared to RT-PCR, offers much better analytical sensitivity. Such increased sensitivity is definitely crucial for identifying viruses in aerosols.

The study's findings provided a wealth of information on the utility of ddPCR, the specificity and sensitivity of RT-PCR and ddPCR assays, as well as recommendation to improve sampling and detection of SARS-CoV-2 virus in air. Indeed, the ddPCR analysis proposed here will improve the currently available analytical strategies and suggest a better approach to monitor the viral load of SARS-CoV-2 in air samples, avoiding false-positive or false-negative results. The exceptional sensitivity of ddPCR enhances the quantitative information of respiratory viruses that are airborne, leading to a more comprehensive understanding of its transmission.

## Data availability statement

The datasets presented in this study can be found in online repositories. The names of the repository/repositories and accession number(s) can be found below: dx.doi.org/10.6084/m9.figshare.24269332.

## Author contributions

RS, RI, NM, and KR: Conceptualization and visualization. RM, RI, and NM: Administration and supervision. RI, KR, NM, RM, RS, JS, NK, NN, MP, SR, RN, and NMH: Preparation of material and data collection. SR, RN, JS, and NMH: Collecting articles and original draft writing. SR, RN, NMH, RI, NM, RS, JS, NK, and NN: Review and editing. All authors read and approved the final manuscript.
